# P-430. Reason for Hospitalization Contrasting Adjudication versus ICD-10 Coding Among Persons with HIV, 2016-2019

**DOI:** 10.1093/ofid/ofae631.630

**Published:** 2025-01-29

**Authors:** Alexander C Commanday, Lindsay Browne, Amanda E Moy, Heather Henderson, Claire E Farel, Darren A Dewalt, Joseph J Eron, Sonia Napravnik

**Affiliations:** School of Medicine, University of North Carolina at Chapel Hill, Chapel Hill, North Carolina, USA, Chapel Hill, North Carolina; UNC Chapel Hill, Chapel Hill, North Carolina; School of Medicine, University of North Carolina at Chapel Hill, Chapel Hill, North Carolina, USA, Chapel Hill, North Carolina; University of North Carolina at Chapel Hill, Chapel Hill, North Carolina; UNC Chapel Hill, Chapel Hill, North Carolina; School of Medicine, University of North Carolina at Chapel Hill, Chapel Hill, North Carolina, USA, Chapel Hill, North Carolina; University of North Carolina at Chapel Hill School of Medicine, Chapel Hill, North Carolina; UNC Chapel Hill, Chapel Hill, North Carolina

## Abstract

**Background:**

Persons with HIV (PWH) have disproportionately high hospitalization rates, the reasons for which are incompletely understood. Establishing hospitalization reasons based on ICD discharge diagnosis codes alone may lead to misclassification. In this study we evaluated the performance of ICD-based reason for hospitalization compared to a newly developed protocol for manual adjudication.

**Figure:**
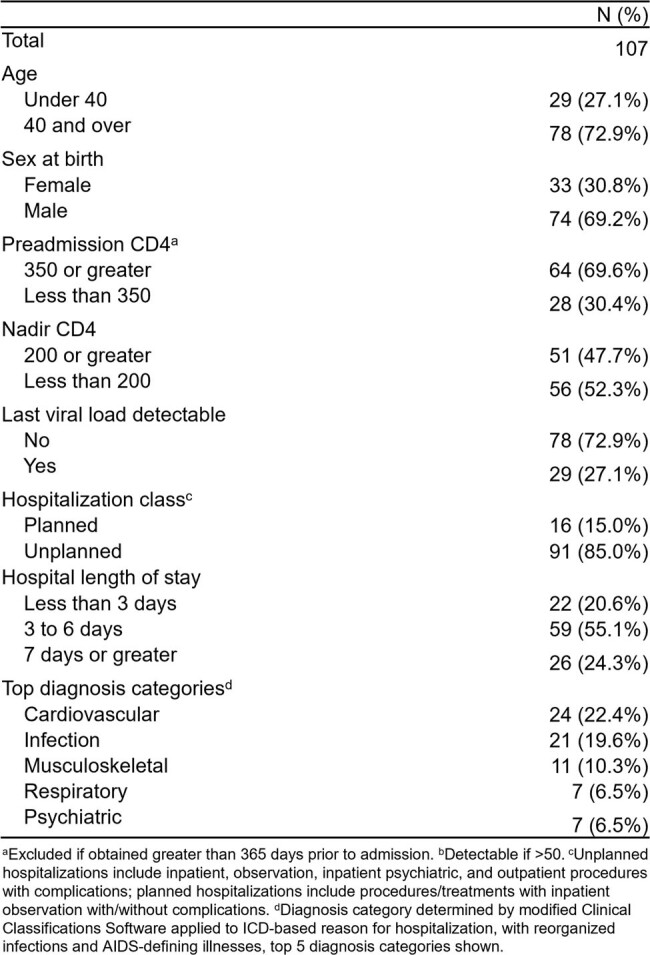

**Methods:**

We randomly sampled 7.3% (N=107) of hospitalizations between 2016 to 2019 among PWH participating in the UNC Center for AIDS Research Clinical Cohort Study (UCHCC). ICD-based reason was defined as the primary ICD-10 discharge diagnosis or secondary discharge diagnosis if primary was HIV and assigned a diagnosis category. A protocol for adjudicating the primary reason for hospitalization was developed. Adjudicated results were categorized and compared to ICD-based reason, with four possible outcomes: complete agreement (same category and diagnosis), partial agreement (same category, different diagnosis), disagreement (different category and diagnosis), and ICD-10 codes inadequate (no available ICD-10 code that captures the complexity and/or nuance of reason for hospitalization).

**Figure:**
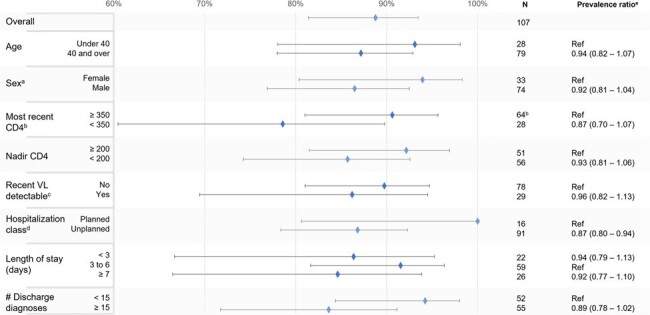

**Results:**

Among the 107 hospitalized PWH, the median age was 51 years (IQR, 37-57), 69.2% were male, and median CD4 count was 672 (IQR, 284-876) (Table 1). Adjudication completely agreed with ICD-based reason in 80 (74.8%) hospitalizations, partially agreed in 15 (14.0%), disagreed in 7 (6.5%), and ICD codes were inadequate in 5 (4.7%). Factors with higher proportion of disagreement or inadequate ICD codes compared to complete or partial agreement were older age, male sex at birth, lower CD4 count, > 15 discharge diagnoses, and longer or unplanned hospitalization (Figure 1).

**Conclusion:**

ICD codes performed well in capturing reason for hospitalization in PWH but were inadequate or disagreed with adjudication in 11.2% of hospitalizations. Patient demographic and clinical factors which reflect the length and complexity of hospitalization may help predict ICD inaccuracy. This work can be expanded to construct an algorithm to determine when adjudication is needed to supplement ICD code review to improve the accuracy of determining reasons for hospitalization in PWH.

**Disclosures:**

**Joseph J. Eron, MD**, Gilead Sciences: Advisor/Consultant|Gilead Sciences: Grant/Research Support|Merck & Co: Advisor/Consultant|ViiV Healthcare: Advisor/Consultant|ViiV Healthcare: Grant/Research Support

